# Nanoemulsions of Cannabidiol, Δ9-Tetrahydrocannabinol, and Their Combination Similarly Exerted Anticonvulsant and Antioxidant Effects in Mice Treated with Pentylenetetrazole

**DOI:** 10.3390/ph18060782

**Published:** 2025-05-23

**Authors:** Pedro Everson Alexandre de Aquino, Francisco Josimar Girão Júnior, Tyciane de Souza Nascimento, Ítalo Rosal Lustosa, Geanne Matos de Andrade, Nágila Maria Pontes Silva Ricardo, Débora Hellen Almeida de Brito, Gabriel Érik Patrício de Almeida, Kamilla Barreto Silveira, Davila Zampieri, Marta Maria de França Fonteles, Edilberto Rocha Silveira, Giuseppe Biagini, Glauce Socorro de Barros Viana

**Affiliations:** 1 Department of Physiology and Pharmacology, Federal University of Ceará, Fortaleza 60430-270, Brazil; pedroeverson.alexandre@gmail.com (P.E.A.d.A.); josimarjunior77@gmail.com (F.J.G.J.); tycianesouza13@gmail.com (T.d.S.N.); italo.rosal@gmail.com (Í.R.L.); gmatos@ufc.br (G.M.d.A.); martafonteles@hotmail.com (M.M.d.F.F.); gbviana@live.com (G.S.d.B.V.); 2 Laboratory of Polymer and Material Innovation, Federal University of Ceará, Fortaleza 60430-270, Brazil; naricard@ufc.br (N.M.P.S.R.); deborahellen@ufc.br (D.H.A.d.B.); erik3gabriel@gmail.com (G.É.P.d.A.); 3 Federal Institute of Education, Science and Technology of Sertão Pernambucano (IF-Sertão PE), Petrolina 56302-100, Brazil; kamilla.barreto@ifsertao-pe.edu.br; 4 Department of Organic and Inorganic Chemistry, Federal University of Ceará, Fortaleza 60430-270, Brazil; davila@ufc.br (D.Z.); edil@ufc.br (E.R.S.); 5 Laboratory of Experimental Epileptology, Department of Biomedical, Metabolic and Neural Sciences, University of Modena and Reggio Emilia, 41125 Modena, Italy

**Keywords:** cannabidiol, epilepsy, nanoemulsion, pentylenetetrazole, Δ9-tetrahydrocannabinol

## Abstract

**Background/Objectives:** The main biologically active molecules of *Cannabis sativa* L. are cannabidiol (CBD) and Δ9-tetrahydrocannabinol (THC). Both exert anticonvulsant effects when evaluated as single drugs, but their possible interaction as components of *C. sativa* extracts has been scarcely studied. For this reason, we evaluated CBD and THC, combined or not, in two seizure models in mice, using an improved vehicle formula. **Methods:** Firstly, acute seizures were induced by intraperitoneal (i.p.) pentylenetetrazole (PTZ, 80 mg/kg), and mice received CBD or THC at 1, 3, 6, and 10 mg/kg, or a CBD/THC 1:1 combination at 1.5, 3, and 6 mg/kg, per os (p.o.), one hour before PTZ administration. Secondly, mice received p.o. CBD (10 mg/kg), CBD/THC (1.5, 3, and 6 mg/kg), valproic acid (50 mg/kg), or vehicle (nanoemulsions without CBD or THC), one hour before PTZ (30 mg/kg, i.p.) every other day for 21 days. Behavioral, biochemical, and immunohistochemical analyses were performed to assess the response to PTZ, oxidative stress, and astroglial activation. **Results:** In the acute model, CBD and THC at 3–10 mg/kg, and their combinations, significantly increased latency to generalized seizures and death, and improved survival rates. In the chronic model, similarly to valproic acid, CBD 10 mg/kg and CBD/THC at 1.5 and 3 mg/kg delayed kindling acquisition, while CBD/THC 6 mg/kg had no effect. CBD and CBD/THC treatments reduced oxidative and nitrosative stress and attenuated astrogliosis, as indicated by decreased glial fibrillary acidic protein and GABA transporter 1 expression and increased inwardly rectifying potassium channel 4.1 expression in hippocampal regions. However, no cannabinoid treatment prevented the impairment in novel object recognition and Y maze tests. **Conclusions:** These findings support the potential role of cannabinoids in counteracting seizures, possibly by reducing oxidative stress and astrogliosis. The study also highlights the importance of nanoemulsions as a delivery vehicle to enhance cannabinoid effectiveness while considering the risks associated with direct cannabinoid receptor activation.

## 1. Introduction

Epilepsy is a chronic neurological condition caused by abnormal and hypersynchronous brain activity, leading to recurring and unpredictable epileptic seizures. The prevalence of active epilepsy worldwide, disregarding cases in remission, is 6.38 per 1000 people [1].

Epilepsy treatment with *Cannabis* sp. and related compounds has drawn increasing attention from researchers in recent years. Studies indicate that certain compounds from *Cannabis*, such as cannabidiol (CBD), a major non-psychoactive cannabinoid, and Δ9-tetrahydrocannabinol (THC), known for its psychoactive effects, both possess antiepileptic properties, including evidence of a possible role in the treatment of pharmacoresistant epilepsy. Particularly, CBD has demonstrated antiepileptic effects in multiple preclinical and clinical studies [2]. The efficacy of CBD in treatment-resistant epilepsy has been confirmed in randomized clinical trials, which led to the FDA approval of Epidiolex^®^ for Dravet and Lennox-Gastaut syndromes [3]. In turn, THC has shown synergistic effects when combined with CBD, improving seizure control in pediatric drug-resistant epilepsy [3].

Nonetheless, large interindividual variability in response to cannabinoids remains a challenge in the pharmacological modulation of the endocannabinoid system for epilepsy. Additionally, the psychoactive effects of THC, related to cannabinoid receptor 1 (CB1) agonism, restrict its clinical use. Altogether, this emphasizes the need for further studies to better understand the potential role of cannabinoids in epilepsy treatment [4].

Despite their therapeutic potential, cannabinoids such as CBD and THC suffer from low oral bioavailability due to poor aqueous solubility, extensive first-pass metabolism, and variable absorption profiles [5]. Among the various nanosystems employed for drug delivery, oil-in-water nanoemulsions stabilized by amphiphilic block copolymers and prepared via probe ultrasonication represent a promising strategy due to their ability to encapsulate hydrophobic active compounds, thereby enhancing their bioavailability, in addition to offering advantages such as cost-effectiveness, physical stability, and a non-toxic profile [6]. Nanoemulsions have emerged as a promising drug delivery system capable of enhancing cannabinoid solubility, protecting them from degradation, and improving systemic absorption. These features are particularly relevant in preclinical epilepsy models, where rapid and consistent brain delivery is crucial to evaluate the anticonvulsant effects [7]. Thus, the development of cannabinoid-loaded nanoemulsions aims to overcome formulation challenges and optimize therapeutic efficacy.

Astrocytes are essential for maintaining the blood–brain barrier and supporting synaptic function, contributing to neurotransmitter homeostasis by clearing glutamate and γ-aminobutyric acid (GABA) from the synaptic cleft [8,9]. This regulation plays a key role in balancing excitatory and inhibitory neurotransmission, with the glutamate–GABA–glutamine cycle being critical for synaptic signaling over time [10].

Under pathological conditions such as infection, trauma, ischemia, hemorrhage, or seizures, astrocytes become activated along with microglia and infiltrating leukocytes, and all of them can secrete inflammatory mediators [11,12]. The consequent inflammatory response triggers reactive astrogliosis, a process characterized by morphological and metabolic changes in astrocytes and altered protein expression, which has been observed in epileptic foci [13,14]. One of the most well-known proteins modified in reactive astrocytes is glial fibrillary acidic protein (GFAP), a component of the astrocytic cytoskeleton and a key molecular marker of astrogliosis. Several other proteins involved in ion homeostasis and neurotransmitter reuptake also undergo expression changes in epilepsy, including potassium channels [14].

The inwardly rectifying potassium channel 4.1 (Kir4.1) is predominantly expressed in astrocytes and plays a key role in regulating extracellular potassium levels [15]. By buffering the extracellular potassium increase during neuronal activity, Kir4.1 prevents excessive depolarization and supports the physiological function of neural networks [16]. Evidence has emerged that endocannabinoids can modulate Kir4.1, independently of CB1 or CB2 receptor activation. This may alter astrocytic K⁺ buffering capacity, impacting neuronal excitability and seizure susceptibility [17,18].

The major GABA transporter-1 (GAT1) is responsible for GABA reuptake into nerve terminals and astrocytes, maintaining inhibitory neurotransmission [19]. While GAT1 expression is higher in GABAergic nerve terminals, it is also present in astrocytes, playing a vital role in synaptic inhibition [20,21]. Although there is no direct experimental data linking cannabinoids to GAT-1 regulation in the provided literature, the endocannabinoid system is known to influence GABAergic transmission, suggesting a potential indirect effect that warrants further investigation [22].

Both Kir4.1 and GAT1 are essential for neuronal communication, and their dysfunction has been implicated in the pathophysiology of epilepsy and other neurological disorders. Current research seeks to further elucidate their roles and potential as therapeutic targets in epilepsy [21,22].

Based on this background, we characterized the effects of different doses of CBD and THC, administered individually or in combination, in two seizure models: (i) pentylenetetrazole (PTZ)-induced acute seizures and (ii) PTZ-induced kindling. Kindling is widely used to mimic epileptogenesis, as repeated administration of subconvulsive doses of PTZ initially causes mild behavioral changes but eventually leads to progressive worsening of motor seizures, with the contribution of oxidation as a pathophysiological mechanism [23,24]. In this manner, we aimed to investigate the modulation of astrocytic Kir4.1 and GAT1 expression as potential factors involved in the antiseizure effects of CBD and THC, loaded in Licuri oil nanoemulsion, and their possible impact on GFAP expression.

## 2. Results

### 2.1. Characterization of Licuri Oil

Licuri oil, analyzed by CG-MS, was composed of fatty acids shown (%) in Table 1. The analysis revealed that licuri oil molar composition consisted of 80.48% saturated fatty acids and 19.52% unsaturated fatty acids. The major compound was lauric acid (42.13%). Approximate values have been found in a recent study [25].

### 2.2. Particle Size and Zeta Potential Measurement

**Figure 1:**
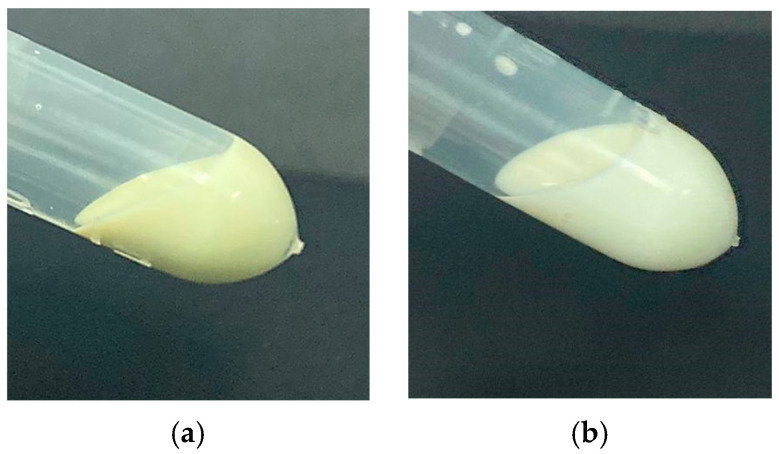
Δ9-tetrahydrocannabinol (THC) and cannabidiol (CBD) nanoemulsions are shown in (**a**) and (**b**), respectively. Note that THC and CBD nanoemulsions were consistent.

The THC and CBD nanoemulsions (Figure 1) visually showed a homogeneous aspect.

The characterization outcomes for nanoemulsions revealed that droplets fell within the nanoemulsion scale [26,27,28], as illustrated in Table 2. The average size of the droplets in the THC-based nanoemulsion was recorded at 237.5 ± 1.457 nm, while for the CBD-based nanoemulsion, it was 247.2 ± 1.153 nm.

The polydispersity index (PDI) is a unitless indicator ranging from 0 to 1.0, reflecting the distribution of particle sizes within a sample. Lower PDI values indicate greater uniformity. In this study, the PDI values obtained were below 0.1, suggesting highly monodisperse emulsions, as shown in Table 2.

As for the zeta potential (ZP), the nanoemulsions exhibited values ranging from −33.9 to −32.4 mV, which categorizes them as stable. Stability increases with the magnitude of the surface charge on the droplets, as higher charges lead to stronger repulsive forces between droplets, mitigating the risk of destabilization. In this study, despite utilizing a non-ionic surfactant, the observed negative ZP values could stem from the attachment of hydroxyl ions (OH⁻) at the oil–water boundary, facilitated by the hydrogen bonding of the ethylene oxide segments of the Pluronic^®^ F127.

### 2.3. Effects of Combined or Separately Administered Cannabidiol (CBD) and Δ9-Tetrahydrocannabinol (THC) on Pentylenetetrazole (PTZ)-Induced Acute Seizures

**Figure 2:**
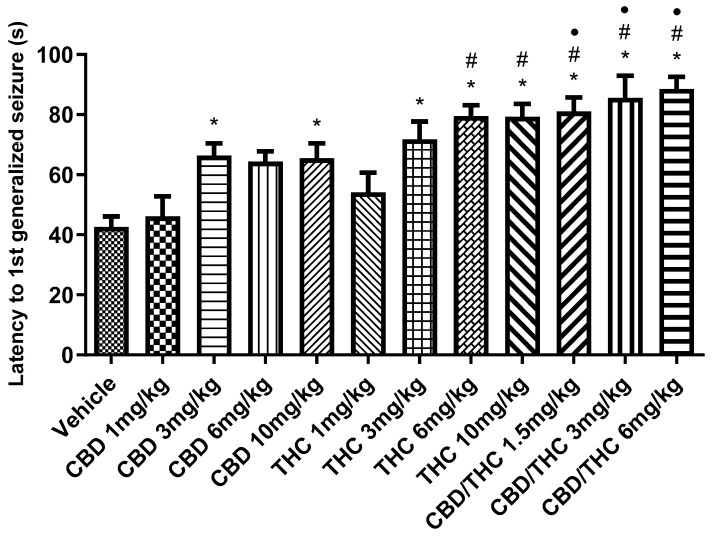
Animals (n = 15 per group) treated with nanoemulsions containing cannabidiol (CBD), Δ9-tetrahydrocannabinol (THC), or a CBD/THC combination (1:1) showed a significant increase in the latency to the first generalized seizure compared to the vehicle group. This increase was more pronounced at intermediate and high doses (3, 6, and 10 mg/kg). The lowest doses (1 mg/kg) of isolated CBD and THC did not show a significant difference. Treatments were administered by gavage, and 60 min later, the animals received intraperitoneal pentylenetetrazole (PTZ, 80 mg/kg). Values are in seconds (s), mean ± standard error of the mean. * *p* < 0.05 compared to the vehicle, # *p* < 0.05 compared to CBD 1 mg/kg, • *p* < 0.05 compared to THC 1 mg/kg. One-way analysis of variance followed by Tukey’s post hoc test.

In the acute PTZ model of generalized tonic–clonic seizures, we analyzed latency to the first generalized seizure and survival. An increase in the latency to the first generalized seizure was observed with intermediate and high doses of CBD and THC (3 mg/kg, 6 mg/kg, and 10 mg/kg), as well as with the CBD/THC (1:1) combination at doses of 1.5 mg/kg, 3 mg/kg, and 6 mg/kg, compared to the vehicle-treated group (illustrated in Figure 2). No significant effect was observed with the lowest dose (1 mg/kg) of CBD or THC separately administered.

Statistical analysis of the data revealed a significant difference between the CBD/THC combinations (1.5 mg/kg, 3 mg/kg, and 6 mg/kg) and the cannabinoids administered individually, especially at intermediate and high doses. The CBD/THC combinations were more effective in increasing both the latency to the first seizure and time of survival, outperforming the effects of the lower doses of isolated CBD and THC. This was indicated by the additional symbols in the graphs (# compared to CBD 1 mg/kg and • compared to THC 1 mg/kg), suggesting that the higher dose combinations provided even greater protection.

**Figure 3:**
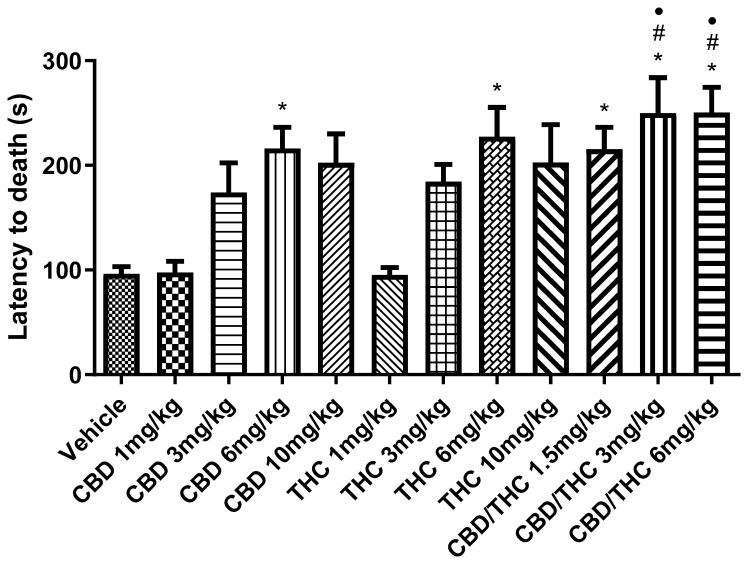
Animals (n = 15 per group) treated with nanoemulsions containing cannabidiol (CBD), Δ9-tetrahydrocannabinol (THC), or a CBD/THC combination (1:1) showed a significant increase in the survival time, measured as latency to death. This increase was more pronounced at intermediate and high doses (3, 6, and 10 mg/kg). The lowest doses (1 mg/kg) of isolated CBD and THC showed no significant difference. Treatments were administered by gavage, and 60 min later, the animals received intraperitoneal pentylenetetrazole (PTZ, 80 mg/kg). Values are in seconds (s), mean ± standard error of the mean. * *p* < 0.05 compared to the vehicle, # *p* < 0.05 compared to CBD 1 mg/kg, • *p* < 0.05 compared to THC 1 mg/kg. One-way analysis of variance followed by Tukey’s post hoc test.

The second parameter, time of survival preceding death, followed a similar trend (Figure 3). The CBD/THC combination at 3 mg/kg and 6 mg/kg resulted in a significant increase in the survival time preceding death, compared to the vehicle-treated group and the lower doses of singularly administered cannabinoids. Notably, the survival rate increased to 20% at the two highest doses of the CBD/THC combination (3 mg/kg and 6 mg/kg).

### 2.4. Effect of Cannabidiol (CBD) and CBD/Δ9-Tetrahydrocannabinol (THC) Combination on the Brain Content of Nitrate/Nitrite in the Pentylenetetrazole (PTZ) Model of Acute Seizures

**Figure 4:**
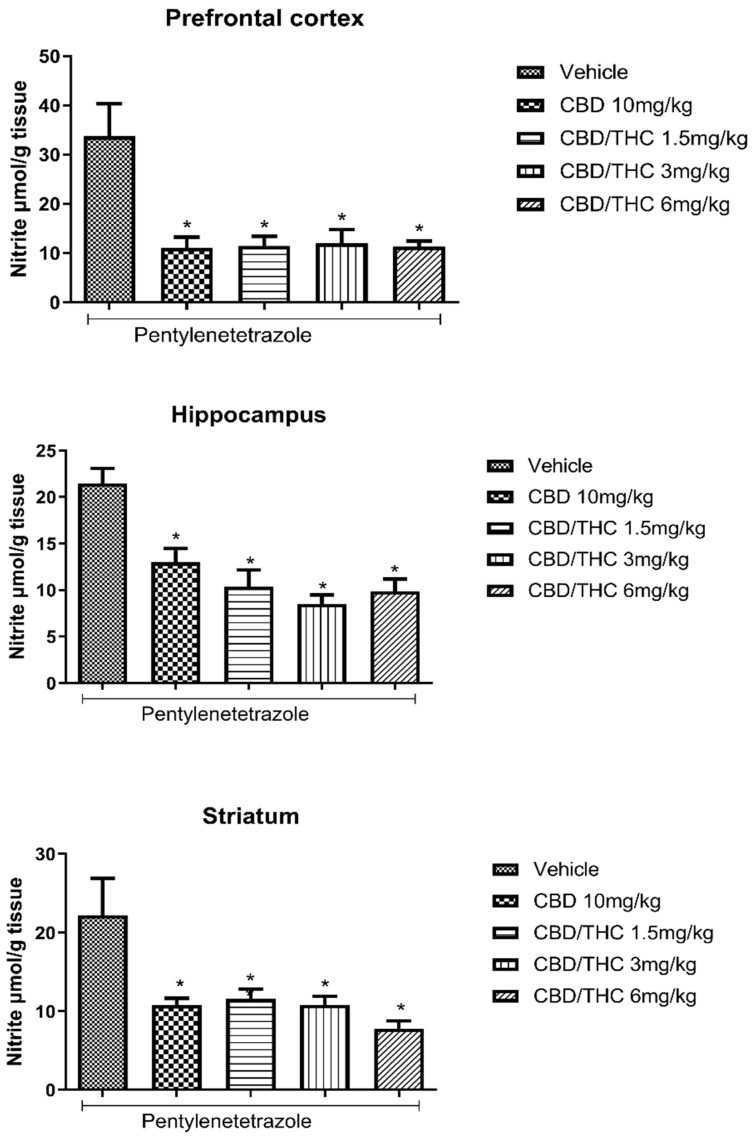
Cannabinoid pretreatment attenuated the increase in the nitrate (NO^3−^)/nitrite (NO^2−^) concentration associated with pentylenetetrazole (PTZ)-induced acute seizures at all tested doses. Animals (n = 15 per group) were gavaged with either vehicle or a nanoemulsion of cannabidiol (CBD) 10 mg/kg or CBD/Δ9-tetrahydrocannabinol (THC) (1:1) at 1.5, 3, or 6 mg/kg. Sixty minutes later, animals were given intraperitoneal PTZ 80 mg/kg. After death, nitrate/nitrite concentrations were measured in the prefrontal cortex, hippocampus, and striatum. Values of nitrite in μmol per gram of tissue are presented as mean ± standard error of the mean. Significant values: * *p* < 0.05 versus vehicle. One-way analysis of variance followed by Tukey’s post hoc test.

We also evaluated nitrosative stress by measuring NO^3−^ and NO^2−^ concentration using the Griess reaction. A significant increase in NO^3−^/NO^2−^ concentration associated with PTZ-induced seizures was seen in three evaluated brain areas: prefrontal cortex, hippocampus, and striatum. Treatment with CBD 10 mg/kg and CBD/THC (1:1) at 1.5, 3, and 6 mg/kg significantly attenuated the increase in NO^3−^/NO^2−^ concentration caused by PTZ-induced acute seizures, as measured in the vehicle-treated group at all doses tested (Figure 4).

### 2.5. Effect of Cannabidiol (CBD) and CBD/Δ9-Tetrahydrocannabinol (THC) Combination on Lipid Peroxidation in the Brain of Pentylenetetrazole Acutely Treated Mice

**Figure 5:**
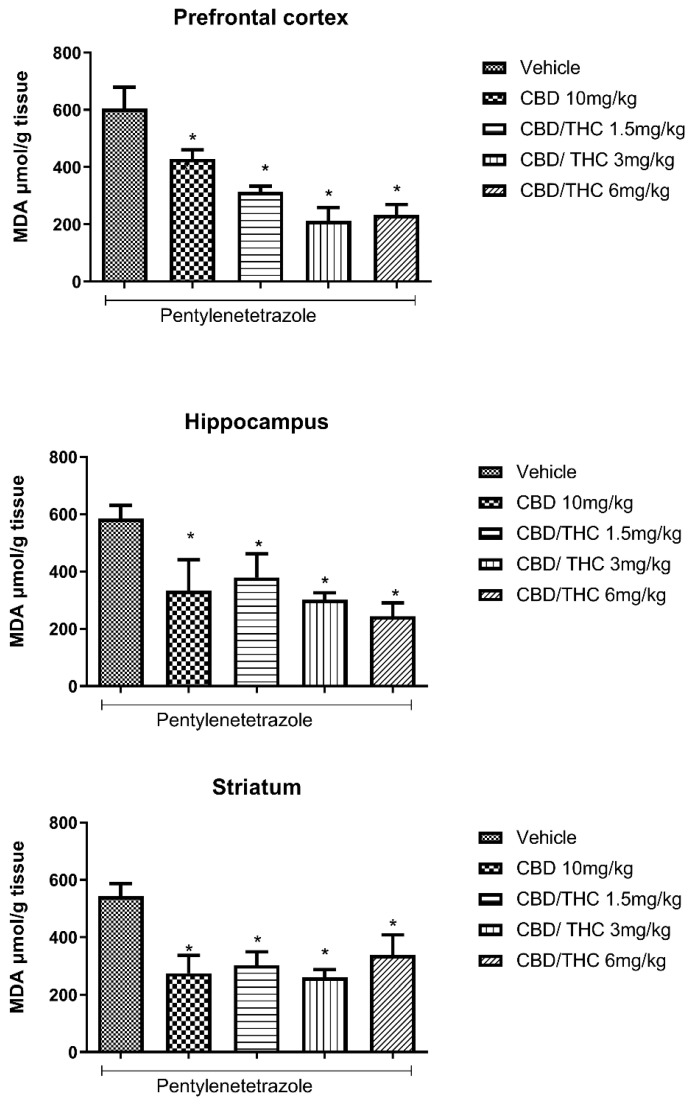
Cannabinoid pretreatment attenuated the increase in malondialdehyde (MDA) concentration associated with pentylenetetrazole (PTZ)-induced acute seizures at all tested doses. Animals (n = 15 per group) were gavaged with either vehicle or nanoemulsion of cannabidiol (CBD) 10 mg/kg or CBD/Δ9-tetrahydrocannabinol (THC) (1:1) at 1.5, 3, or 6 mg/kg. Sixty minutes later, animals were given intraperitoneal PTZ at 80 mg/kg. After death, MDA concentration was measured in the prefrontal cortex, hippocampus, and striatum. Values of MDA in μmol per gram of tissue are presented as mean ± standard error of the mean. Significant values: * *p* < 0.05 versus vehicle. One-way analysis of variance and Tukey’s post hoc test.

Lipid peroxidation is a consequence of oxidative stress induced by the action of reactive oxygen species on lipids composing the cell membrane and membranous organelles. Lipid peroxidation in biological samples can be indicated by the concentration of its main stable end product, that is, malondialdehyde (MDA), which can be measured by the thiobarbituric acid–reactive substance (TBARS) assay. A significant increase in MDA caused by PTZ-induced acute seizures was evidenced in all the evaluated brain areas (prefrontal cortex, hippocampus, and striatum). Pretreatment with CBD 10 mg/kg and CBD/THC (1:1) at 1.5, 3, and 6 mg/kg significantly attenuated the increase in MDA concentration associated with PTZ-induced acute seizures, compared to the vehicle-treated group at all doses tested (Figure 5).

### 2.6. Effects of Cannabidiol (CBD) and CBD/Δ9-Tetrahydrocannabinol (THC) Combination on Pentylenetetrazole (PTZ)-Induced Kindling

**Figure 6:**
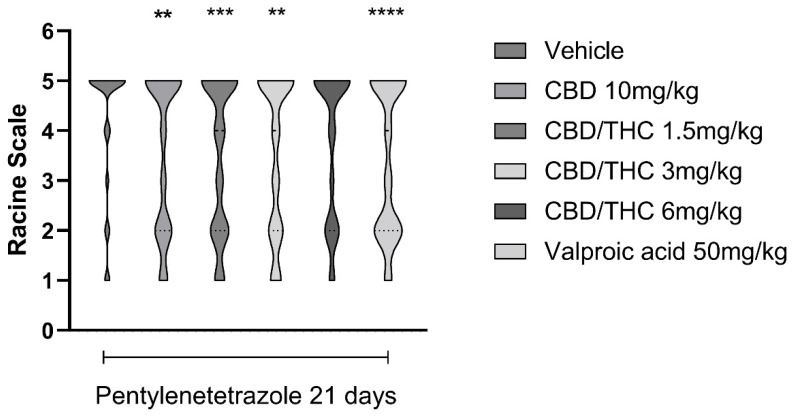
Pretreatment with per os cannabidiol (CBD) 10 mg/kg or a CBD/Δ9-tetrahydrocannabinol (THC) (1:1) combination at 1.5 and 3 mg/kg significantly slowed the development of pentylenetetrazole (PTZ)-induced kindling. Every other day for 21 days, animals (n = 15 per group) were gavaged with either vehicle, a nanoemulsion of CBD 10 mg/kg or CBD/THC 1:1 1.5, 3, or 6 mg/kg, or valproic acid 50 mg/kg. Sixty minutes later, animals (n = 15 per group) were given PTZ 30 mg/kg (i.p.). After the last PTZ administration, seizures were staged according to the Racine scale: stage 1—normal behavior; stage 2—hyperactivity; stage 3—repeated vertical movements that may represent stereotypical behavior; stage 4—clonus of the front paws and “rearing”; and stage 5—clonus of the four paws, loss of the righting reflex and fall, tonic ventral flexion of the head and the whole body, and tonic hind paw extension. Results are expressed as median values. Significant values: ** *p* < 0.01; *** *p* < 0.001; **** *p* < 0.0001 versus vehicle. Kruskal Wallis’ and Dunn’s post hoc test.

Intraperitoneal (i.p.) administration of PTZ (30 mg/kg) every other day over 21 days gradually increased the seizure stage. Pretreatment with CBD 10 mg/kg, the CBD/THC (1:1) combination at 1.5 and 3 mg/kg, or valproic acid 50 mg/kg significantly delayed the appearance of more severe seizures following the onset of PTZ treatment. In contrast, CBD/THC at 6 mg/kg was not able to prevent the development of full kindling (Figure 6).

### 2.7. Changes in Behavioral Test Performance of Cannabidiol (CBD) and CBD/Δ9-Tetrahydrocannabinol (THC) Combination-Treated Mice in Consequence of Pentylenetetrazole (PTZ)-Induced Kindling

**Figure 7:**
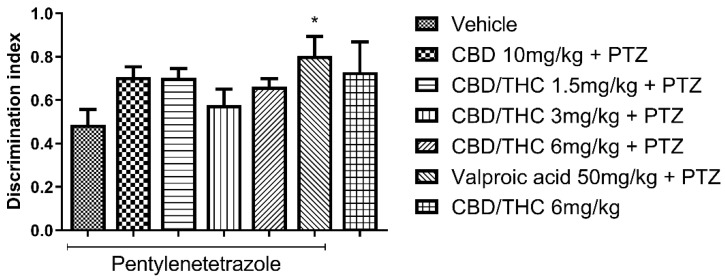
Pretreatment with per os cannabidiol (CBD) 10 mg/kg or a CBD/Δ9-tetrahydrocannabinol (THC) (1:1) combination at 1.5 and 3 mg/kg did not prevent impairment in the novel object recognition test induced by chronic pentylenetetrazole (PTZ) administration for kindling. Every other day for 21 days, animals (n = 15 per group) were gavaged with either vehicle, a nanoemulsion of CBD 10 mg/kg or CBD/THC (1:1) at 1.5, 3, or 6 mg/kg, or valproic acid 50 mg/kg. Sixty minutes later, animals were given intraperitoneal PTZ at 30 mg/kg. One group was given CBD/THC (1:1) 6 mg/kg without PTZ. On the day after the last drug administration, behavioral tests were carried out. The discrimination index is presented as mean ± SEM. Significant values: * *p* < 0.05. One-way analysis of variance followed by Tukey’s post hoc test.

In the novel object recognition test (ORT) for short-term memory, pretreatment with CBD 10 mg/kg and CBD/THC (1:1) combination (1.5, 3, and 6 mg/kg) did not differ significantly from pretreatments with vehicle or valproic acid (50 mg/kg). On the other hand, pretreatment with valproic acid (50 mg/kg) significantly prevented short-term memory impairment compared to pretreatment with vehicle (*p* < 0.05), as shown in Figure 7. Furthermore, treatment with CBD/THC (1:1) (6 mg/kg) without PTZ produced a mean value similar to that observed in the vehicle-pretreated group. These findings show that the cannabinoid pretreatments failed to prevent short-term memory impairment, while precluding a definitive statement on the effect of cannabinoids on short-term memory in this model.

**Figure 8:**
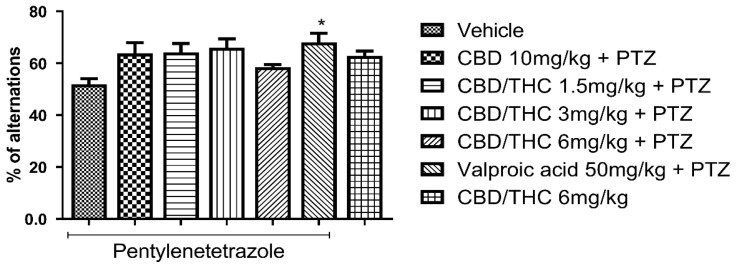
Effect of per os cannabidiol (CBD) 10 mg/kg or a CBD/Δ9-tetrahydrocannabinol (THC) (1:1) combination at 1.5 and 3 mg/kg on Y-maze test performance in pentylenetetrazole (PTZ)-kindled mice. No cannabinoid preemptive treatment prevented the impairment in Y-maze test performance related to PTZ-induced kindled seizures. Every other day for 21 days, animals (n = 15 per group) were gavaged with either vehicle, a nanoemulsion of CBD 10 mg/kg or CBD/THC (1:1) combination at 1.5, 3, or 6 mg/kg, or valproic acid 50 mg/kg. After 60 min, animals were given intraperitoneal PTZ 30 mg/kg. One group was given CBD/THC (1:1) 6 mg/kg without PTZ. On the day after the last drug administration, behavioral tests were carried out. The percentage of arm alternation is presented as mean ± SEM. Significant values: * *p* < 0.05. One-way analysis of variance followed by Tukey’s post hoc test.

We also evaluated behavioral performance in the Y-maze test, which assesses short-term and working memory. Similar to the ORT results, pretreatment with CBD 10 mg/kg and CBD/THC (1:1) combination (1.5, 3, and 6 mg/kg) did not differ significantly from the pretreatment groups with vehicle or valproic acid (50 mg/kg). However, pretreatment with valproic acid (50 mg/kg) significantly prevented short-term and working memory impairment compared to pretreatment with vehicle (*p* < 0.05), as shown in Figure 8. Additionally, the group treated with CBD/THC (1:1) (6 mg/kg) without PTZ showed a mean value similar to that of the group pretreated with vehicle. As there was no significant difference between the cannabinoid pretreatments and both vehicle and valproic acid pretreatments, no cognitive enhancement by cannabinoids was shown. Nonetheless, a conclusive statement regarding the effect of cannabinoid pretreatments in this paradigm cannot be made.

### 2.8. Effects of Cannabidiol (CBD) and CBD/Δ9-Tetrahydrocannabinol (THC) Combination on Body Weight

**Figure 9:**
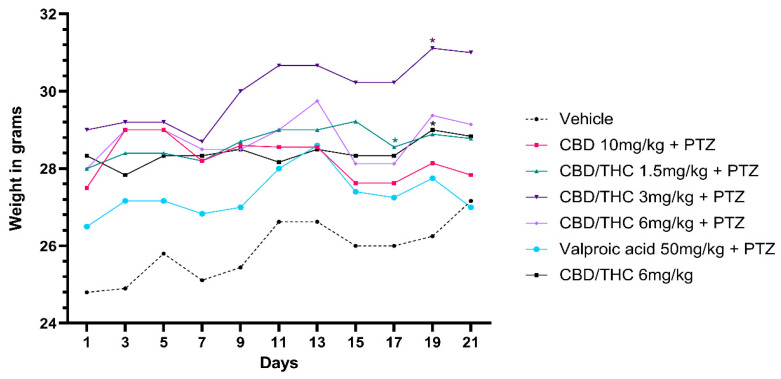
Effect on body weight of per os cannabidiol (CBD) 10 mg/kg or a CBD/Δ9-tetrahydrocannabinol (THC) (1:1) combination at 1.5 and 3 mg/kg, their vehicle, or valproic acid, in presence of pentylenetetrazole (PTZ)-induced kindling. Every other day for 21 days, animals (n = 15 per group) were gavaged with either vehicle, a nanoemulsion of CBD 10 mg/kg or CBD/THC (1:1) at 1.5, 3, or 6 mg/kg, or valproic acid 50 mg/kg. Sixty minutes later, animals were given intraperitoneal PTZ 30 mg/kg. Results are expressed as median values. Significant values: * *p* < 0.05 versus vehicle. Kruskal–Wallis test followed by post hoc Dunn’s test.

We investigated the effects of cannabinoid treatment on the body weight of mice subjected to PTZ-induced kindling, as well as in non-kindled mice. Mice receiving preemptive intragastric treatment with a 1:1 CBD/THC combination at doses of 1.5 and 3 mg/kg exhibited a statistically significant increase in body weight after the 21-day PTZ-induced kindling protocol compared to the vehicle-treated group. Similarly, non-kindled mice treated with 6 mg/kg of the CBD/THC (1:1) combination also showed increased body weight. In contrast, no significant differences in body weight were observed in mice treated with CBD 10 mg/kg, CBD/THC (1:1) 6 mg/kg, or valproic acid 50 mg/kg followed by PTZ, when compared to the vehicle-treated group (Figure 9).

### 2.9. Effects of Cannabidiol (CBD) and CBD/Δ9-Tetrahydrocannabinol (THC) Combination on Astrocyte Activation and After Pentylenetetrazole (PTZ)-Induced Kindling

**Figure 10:**
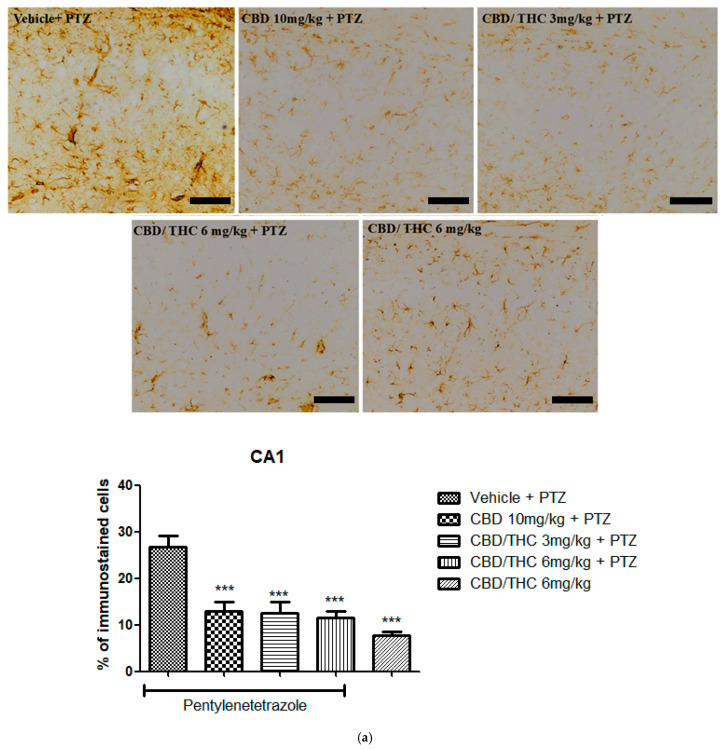

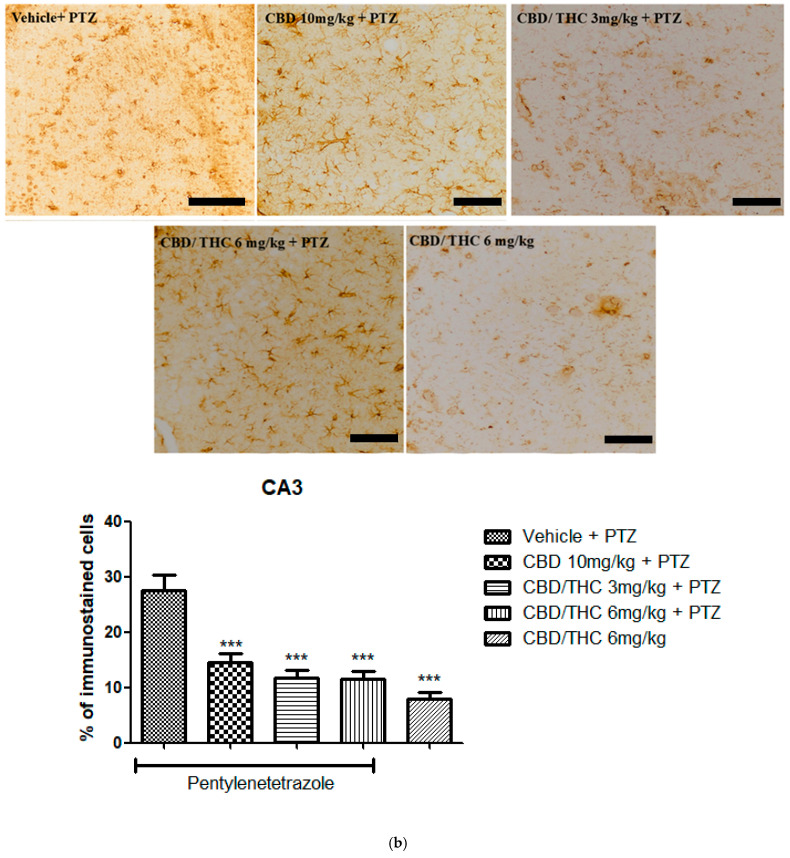

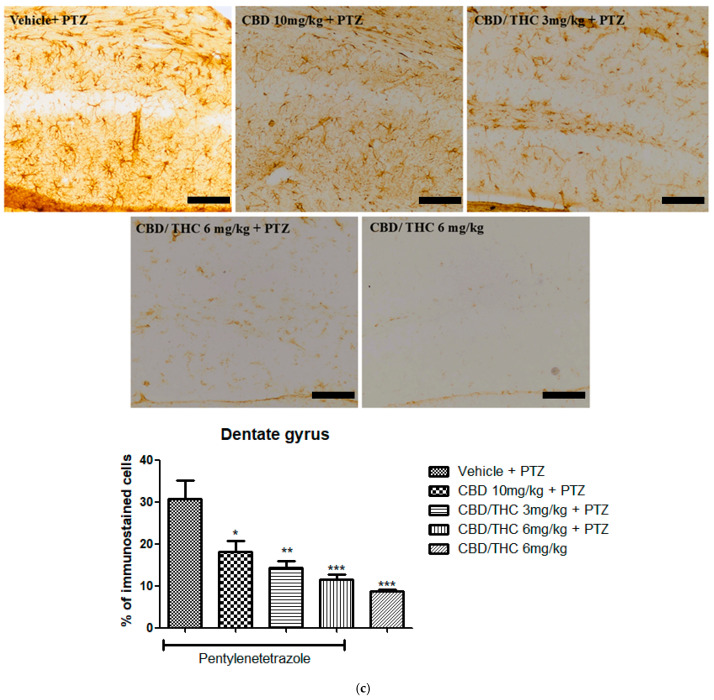
Cannabinoid pretreatment prevented changes in glial fibrillary acidic protein (GFAP) immunoreactivity in 3 hippocampal areas (CA1—(**a**); CA3—(**b**); and dentate gyrus—(**c**)), as observed in pentylenetetrazole (PTZ)-treated mice receiving the cannabinoid vehicle. Every other day for 21 days, animals were gavaged with either vehicle or a nanoemulsion of cannabidiol (CBD) 10 mg/kg or a CBD/Δ9-tetrahydrocannabinol (THC) (1:1) combination at 1.5 or 3 mg/kg. After 60 min, animals were given PTZ 30 mg/kg (i.p.). One group was given CBD/THC (1:1) 6 mg/kg without PTZ. On the day after the last drug administration, after behavioral testing, animals were euthanized and transcardially perfused for immunohistochemistry (4 animals per group). One-way analysis of variance followed by Tukey’s post hoc test. Significant values: * *p* < 0.05; ** *p* < 0.01; *** *p* < 0.001 versus vehicle. Calibration bars, 100 µm.

Immunohistochemical analysis revealed significant alterations in the expression of GFAP, GAT-1, and Kir4.1 across the hippocampal regions, CA1, CA3, and dentate gyrus, following cannabinoid pretreatment. Immunoreactivity for GFAP was significantly preserved in PTZ-kindled animals treated with CBD (10 mg/kg) or CBD/THC (1:1) (3 and 6 mg/kg, intragastric) compared to the vehicle-treated group, in which astrocyte reactivity was remarkable, as confirmed by the lower values observed in the CBD/THC (1:1) (6 mg/kg) group without PTZ, thus suggesting a direct effect of PTZ on astrocytic reactivity (Figure 10).

### 2.10. Effects of Cannabidiol (CBD) and CBD/Δ9-Tetrahydrocannabinol (THC) Combination on Markers of Network Activity After Pentylenetetrazole (PTZ)-Induced Kindling

**Figure 11:**
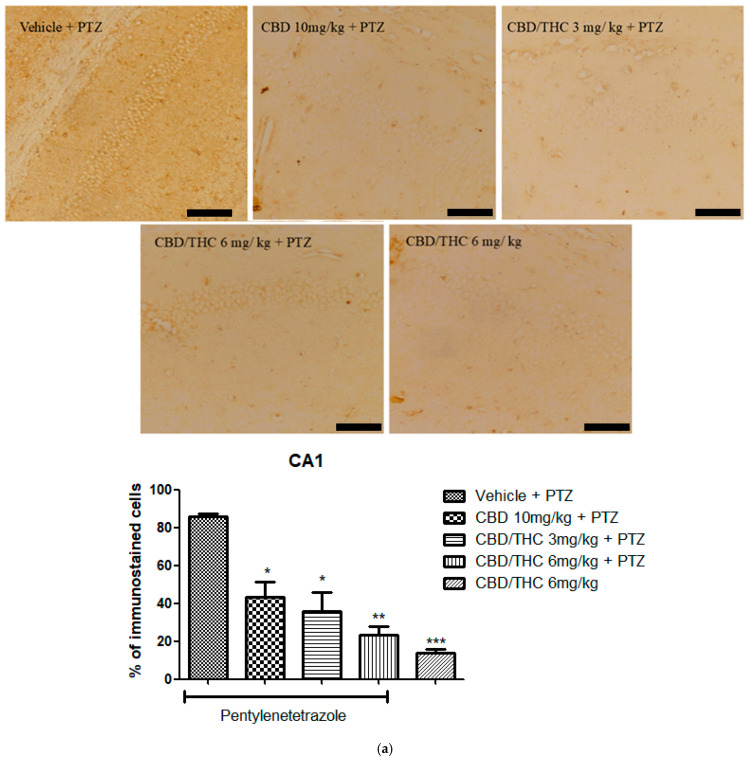

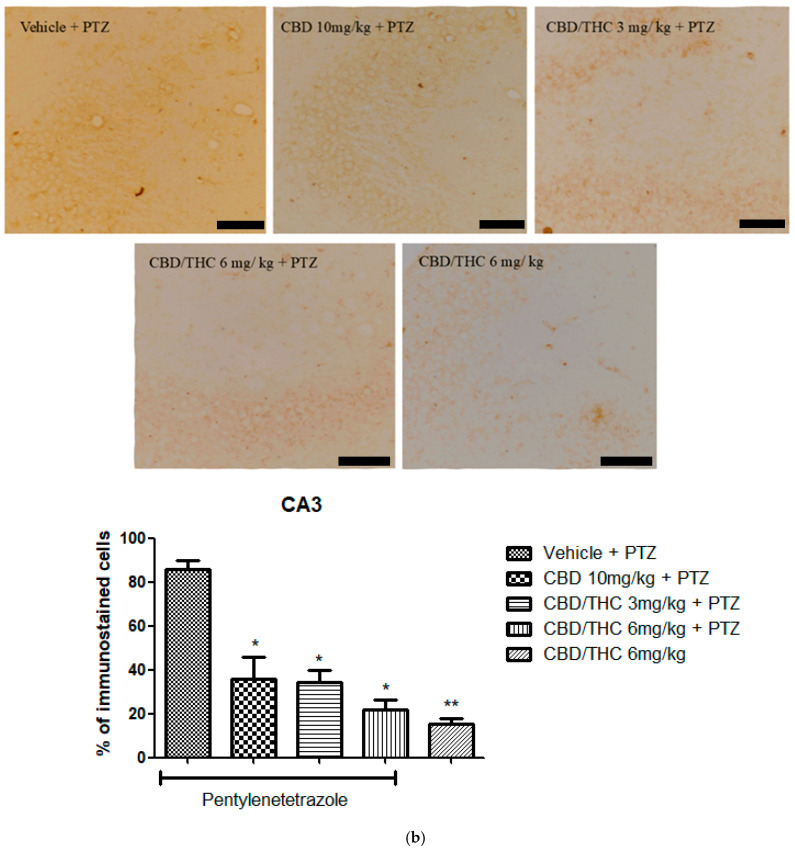

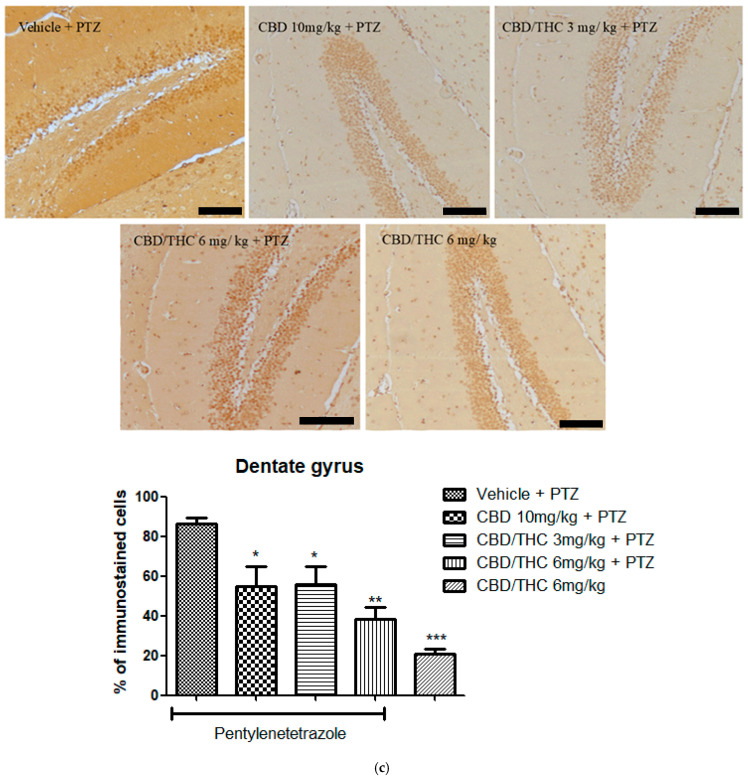
Cannabinoid pretreatment prevented changes in γ-aminobutyric acid (GABA) transporter 1 (GAT1) immunoreactivity in 3 hippocampal areas (CA1—(**a**); CA3—(**b**); and dentate gyrus—(**c**)), as observed in pentylenetetrazole (PTZ)-treated mice receiving the cannabinoid vehicle. Every other day for 21 days, animals were gavaged with either vehicle or a nanoemulsion of cannabidiol (CBD) 10 mg/kg or a CBD/Δ9-tetrahydrocannabinol (THC) (1:1) combination at 1.5 or 3 mg/kg. After 60 min, animals were given intraperitoneal PTZ 30 mg/kg. One group was given CBD/THC (1:1) 6 mg/kg without PTZ. On the day after the last drug administration, after behavioral testing, animals were euthanized and transcardially perfused for immunohistochemistry (4 animals per group). One-way analysis of variance followed by Tukey’s post hoc test. Significant values: * *p* < 0.05; ** *p* < 0.01; *** *p* < 0.001 versus vehicle. Calibration bars, 100 µm.

Similarly to GFAP immunostaining, GAT-1 immunoreactivity was significantly lower in all cannabinoid-treated groups, indicating a potential modulation of GABAergic neurotransmission (Figure 11).

**Figure 12:**
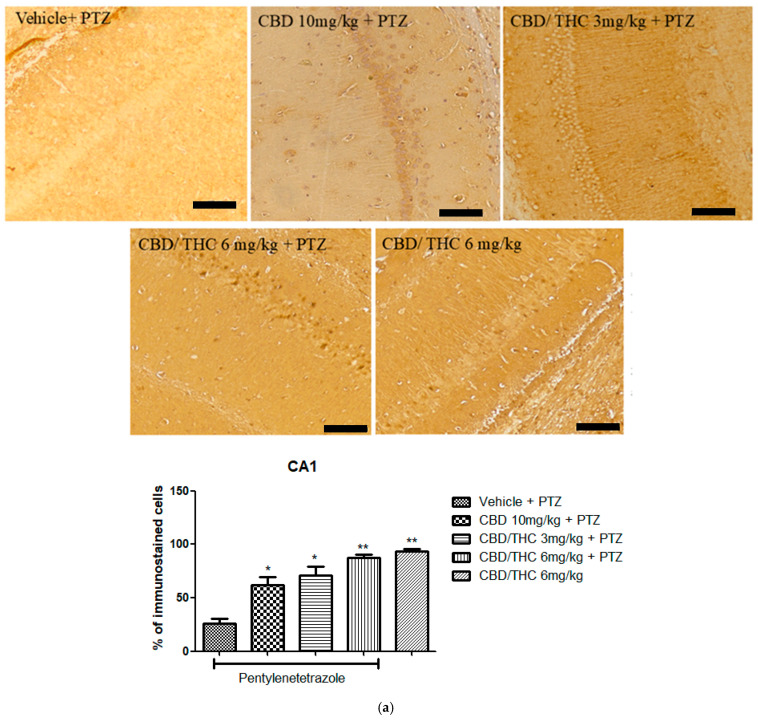

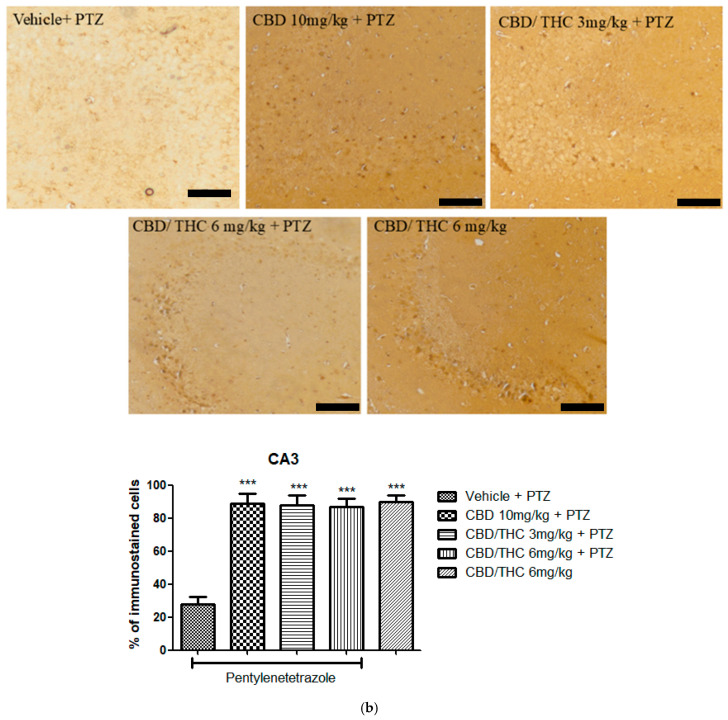

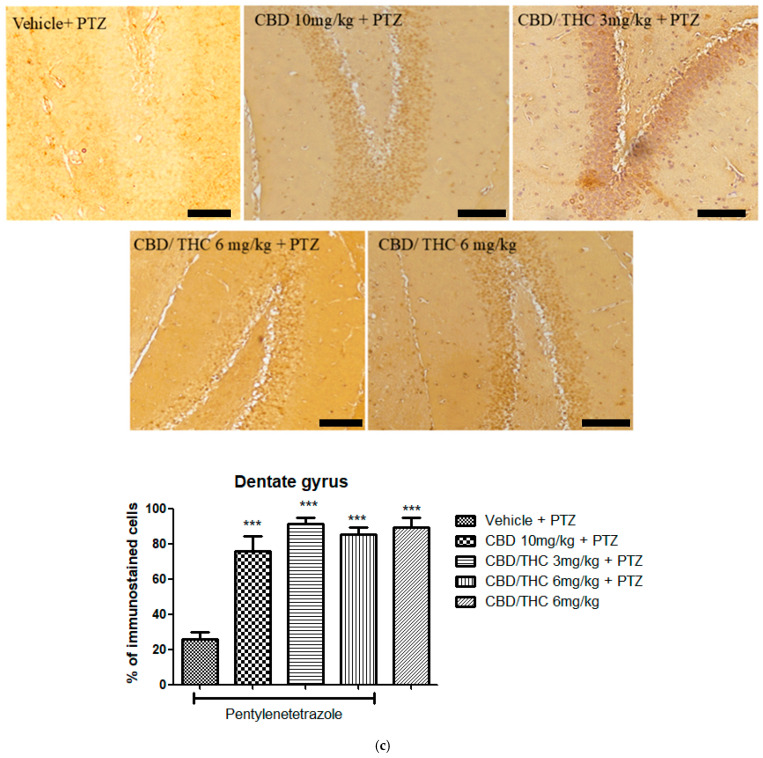
Cannabinoid pretreatment prevented changes in Kyr1.4 immunoreactivity in 3 hippocampal areas (CA1—(**a**); CA3—(**b**); and dentate gyrus—(**c**)), as observed in pentylenetetrazole (PTZ)-treated mice receiving the cannabinoid vehicle. Every other day for 21 days, animals were gavaged with either vehicle or a nanoemulsion of cannabidiol (CBD) 10 mg/kg or a CBD/Δ9-tetrahydrocannabinol (THC) (1:1) combination at 1.5 or 3 mg/kg. After 60 min, animals were given intraperitoneal PTZ 30 mg/kg. One group was given CBD/THC 1:1 6 mg/kg and no PTZ. On the day after the last drug administration, after behavioral testing, animals were euthanized and transcardially perfused for immunohistochemistry (4 animals per group). One-way analysis of variance followed by Tukey’s post hoc test. Significant values: * *p* < 0.05; ** *p* < 0.01; *** *p* < 0.001 versus vehicle. Calibration bars, 100 µm.

In contrast, Kir4.1 immunoreactivity was significantly higher in all cannabinoid-treated groups, including the non-kindled CBD/THC (1:1) (6 mg/kg) group, in all hippocampal regions analyzed (Figure 12). This suggests that PTZ can increase the expression of Kir4.1, and that cannabinoids may play a possible role in preserving potassium homeostasis and neuronal stability.

## 3. Discussion

In this study, we have investigated the effects of CBD and THC nanoemulsions on animal models of acute and chronic seizures induced by PTZ. These nanoemulsions demonstrated efficacy in modulating seizures and attenuating associated brain damage, at concentrations much lower than those reported as successful in other reports [26,27,28]. The nanometric size of the particles in these nanoemulsions suggests an optimized formulation for therapeutic action. In acute models, high doses of CBD, THC, and their combinations significantly prolonged the latency to the first generalized seizure and to death as well, indicating an antiseizure action. These treatments also resulted in decreased levels of oxidative and nitrosative stress markers in brain regions important for seizure onset. In the chronic model, that is, PTZ-induced kindling, preemptive treatment with CBD, lower dose CBD/THC combinations, and valproic acid slowed the progression of kindling acquisition, while a higher dose of CBD/THC showed no efficacy. From a behavioral standpoint, no significant differences were observed in most treated groups, except for those receiving valproic acid, indicating a cognitive deficit possibly related to the epilepsy model that was not addressed by the treatments. Nonetheless, a relationship between the cognitive deficit and the cannabinoid treatment per se cannot be ruled out, given that behavioral deficit was also seen in the group treated with CBD/THC nanoemulsions without PTZ. Furthermore, immunohistochemistry findings indicated neurochemical changes, with a decreased expression of GFAP and GAT1 and an increased expression of Kir4.1 in cannabinoid-treated animals, which may represent possible mechanisms of the disease improvement afforded by the cannabinoid nanoemulsions.

The advantage of nanoemulsions, as demonstrated in this study, lies in their effective absorption and ability to cross biological barriers, including the blood-brain barrier, thereby enhancing the bioavailability of the tested compounds. Nakano et al. [26] developed an innovative nanoemulsion formulation to improve the intestinal absorption of CBD, highlighting the potential of these approaches in epilepsy-related models and other neurological conditions. Additionally, Ahmed et al. [27] created a CBD nanoemulsion for direct nasal-to-brain delivery, enhancing drug bioavailability and addressing the specific organic site of action. Furthermore, El Sohly et al. [28] demonstrated that an UltraShear nanoemulsion of CBD significantly increased its oral–gastrointestinal bioavailability in rats, offering a promising alternative for delivering cannabinoids with enhanced efficacy, thus supporting our findings.

These results are consistent with findings from other researchers who have investigated the effects of *Cannabis* compounds in epilepsy models. For example, Namvar et al. [29] corroborated the effectiveness of a *Cannabis sativa* extract at a dose of 800 mg/kg in rats in modulating PTZ-induced seizures but did not delve deeply into behavioral effects. Additionally, Lu et al. [30] showed that CBD treatment improves epilepsy by acting on metabolism and calcium signaling pathways.

During seizures, an increase in levels of MDA and nitrate/nitrite is observed, which indicates nitrosative and oxidative stress [31]. This increase results from intense neuronal activity in the brain, leading to high oxygen consumption and excessive production of reactive oxygen species and reactive nitrogen species [32,33]. MDA, a byproduct of lipid peroxidation, reflects damage to cell membranes caused by the oxidation of unsaturated fatty acid residues of membrane phospholipids. Simultaneously, the elevation of nitrate/nitrite, derived from nitric oxide metabolism, points to increased nitrosative stress [34,35]. Both oxidative and nitrosative stresses are known to be closely related to neuroinflammation [36,37]. This oxidative environment not only reflects cellular damage caused by seizures but also can contribute to epilepsy progression by altering neuronal and glial function and metabolism, thus increasing susceptibility to future seizures [38]. The present study showed that the levels of oxidative and nitrosative stress markers, MDA, and nitrate/nitrite in the groups pretreated with CBD and THC nanoemulsions were significantly lower than in the vehicle-pretreated group, suggesting that the antioxidative properties of the tested cannabinoids may have contributed to their antiseizure effects to some extent.

Regarding the PTZ-induced kindling model, which emulates chronic epileptogenesis, a delaying effect of CBD and CBD/THC combinations on kindling acquisition was observed, highlighting the potential of these compounds in modifying the progression of epilepsy. The use of *Cannabis* compounds for epilepsy has drawn attention, especially after studies like that of Anderson et al. [39], who emphasized the therapeutic potential of CBD and THC in epilepsy models, including Dravet syndrome. Together, these studies complement and expand on our findings, pointing to several approaches and mechanisms by which *Cannabis* compounds can be useful in treating seizure-related disorders.

CBD has shown beneficial cognitive effects in animal models of epilepsy, as well as neutral or beneficial cognitive effects in humans with epilepsy [40]. Nevertheless, studies such as that by Gáll et al. [41], which analyzed chronic CBD treatment in the PTZ-induced kindling model, found that while CBD increased latency to the first seizure and reduced mortality, behavioral changes such as decreased vertical exploration and discrimination in the novel ORT were also observed. Additionally, CBD has been reported to improve cognitive deficits in models of fetal alcoholic exposure disorder [42], glutamatergic N-methyl-D-aspartate (NMDA) antagonist-induced schizophrenia-like behavior [43], scopolamine-induced memory impairment [44], and Alzheimer’s disease [45], with the latter case associated with a shift from M1 to M2 phenotype of activated hippocampal microglia. Furthermore, a selective agonist of the cannabinoid type 2 (CB2) receptor, which is upregulated in Alzheimer’s disease in humans and animal models, has been shown to improve cognitive deficits in an animal model of the disease [46]. In our study, groups treated with cannabinoid nanoemulsions, even without PTZ, failed to show cognitive improvement. Behavioral tests, including ORT and Y-maze, did not show significant differences in most groups, except for the valproic acid-pretreated group. This is associated with the fact that a sham-kindled group was not included in analyses, preventing the inference of a possible cognitive worsening induced by cannabinoids. Therefore, a definitive assertion on the effect of cannabinoids in this model is not possible.

Our results demonstrate that in mice subjected to PTZ-induced kindling, the expression of GFAP, an astrogliosis marker, is decreased in animals preemptively treated with CBD and THC nanoemulsions relative to those preemptively treated with vehicle. Astrocytes play a critical role in regulating the brain tissue environment, including synaptic activity modulation and neurotransmitter homeostasis. Previous studies, such as those by Mika et al. [47] and Devinsky et al. [48], emphasize the importance of astrocytes in epilepsy pathophysiology, primarily through changes in the expression of proteins like GFAP, which entails dysfunction in glutamate uptake. The reduction in GFAP expression observed in our study may indicate a protective action of the cannabinoid nanoemulsions, possibly through modulation of astrocytic function, contributing to a more stable synaptic environment and reduced susceptibility to seizures.

Previous studies showed that treatment with *Cannabis* sativa extracts resulted in a decrease in GFAP expression in hippocampal astrocytes in rats, suggesting a protective effect in neurological conditions characterized by astrocytic reactivity, reaffirming our findings [49]. The research focused on analyzing the effects of *Cannabis* treatment on GFAP expression, a crucial marker of astrogliosis in the brain’s response to injuries or diseases. The reduction of GFAP expression implies that *Cannabis* compounds may act by modulating glial physiology, potentially protecting neurons from damage caused by excess inflammatory mediators.

Kozela et al. [50] investigated how CBD modulates astrocyte activity in various neurological pathology models, including ischemia, Alzheimer’s-like disease, multiple sclerosis, sciatic nerve injury, epilepsy, and schizophrenia. The results showed that CBD suppressed increased astrocyte reactivity in those conditions, as well as reduced pro-inflammatory activity and signaling in astroglial cells, suggesting its therapeutic potential in mitigating tissue dysfunction in such neurological diseases. Furthermore, Mao et al. [51] explored the neuroprotective effects of CBD in rats with chronic epilepsy, observing a significant reduction in seizure severity, neuronal loss, and astrocyte hyperplasia in the hippocampus.

The present study showed that CBD and the combination of CBD and THC can increase the expression of Kir4.1, which, along with the probable modulation of other ion channels, could influence neuronal excitability. However, these results are preliminary, and a detailed understanding of the mechanisms involved and how cannabinoids affect Kir4.1 from the perspective of seizure and epilepsy pathophysiology remains a subject of research [52].

The GAT-1 transporter plays a crucial role in epilepsy by regulating the reuptake of the inhibitory neurotransmitter GABA at synaptic terminals and glial cells. Its dysfunction is associated with reduced synaptic inhibition, contributing to neuronal hyperexcitability and, consequently, the generation and propagation of epileptic seizures. Modulating GAT-1 activity may offer a potential therapeutic approach to control seizures and improve clinical outcomes in patients with epilepsy [53].

Carvill et al. [54], investigating a type of epilepsy characterized by myoclonic-atonic seizures, identified mutations in the GAT1 encoding gene, SLC6A1, as an etiological factor, leading to a loss of function of the GABA transporter. This study expands the understanding of the genetic basis of epilepsy, offering new insights into diagnostics and treatments focused on GABA regulation, and suggests a significant role of altered GABA reuptake in epilepsy pathophysiology. The increase in GAT1 observed in our vehicle-treated animals most likely represents a pathophysiological element of epileptogenesis, which is restored by cannabinoid treatment. This finding reinforces the importance of GAT-1 in regulating inhibitory neurotransmission and maintaining the excitatory–inhibitory balance in the brain, highlighting its potential as a therapeutic target for specific interventions in epilepsy and paving the way for future investigations into GAT-1 modulation as a strategy to control neuronal hyperexcitability.

Our immunohistochemical findings revealed significant neurochemical alterations in animals that underwent preemptive treatment with CBD and its combination with THC, highlighting a decrease in GAT1 expression. This observation suggests that CBD, alone or in combination with THC, may influence inhibitory neurotransmission, possibly modulating synaptic activity and contributing to restoring an altered excitatory–inhibitory balance in the brain. These results reinforce the therapeutic potential of these compounds in modulating neurological disorders such as epilepsy through the regulation of GAT-1 and Kir4.1.

The findings of this study highlight the therapeutic potential of cannabinoid-based nanoemulsions in modulating epileptic activity, particularly in a chronic PTZ-kindling model. Building on these results, future investigations should prioritize the refinement of the nanoemulsion system, with the aim of achieving smaller and more uniform droplet sizes (<200 nm), improving physicochemical stability, and ensuring batch-to-batch reproducibility. These enhancements are expected to positively impact the pharmacokinetic profile of the formulations, including absorption, distribution, and bioavailability. Moreover, it is crucial to conduct long-term safety evaluations and detailed pharmacodynamics studies, especially regarding the neurodevelopmental and cognitive implications of chronic cannabinoid exposure. Mechanistic studies should also be expanded to investigate how CBD and THC interact with specific molecular targets such as Kir4.1, GAT-1, GFAP, and pro-inflammatory signaling pathways, in order to better understand their roles in seizure modulation, neuroprotection, and glial function. Finally, translational research using more clinically relevant models of epilepsy—such as genetic models, drug-resistant seizures, or comorbid psychiatric disorders—will be essential to support the clinical advancement of these formulations and determine their real-world applicability in complex patient populations.

## 4. Materials and Methods

### 4.1. Plant Material

Crude oil from *Cannabis indica* (CBD chemotype (Cs-CBD)) and *Cannabis sativa* (THC chemotype (Cs-THC)) was produced and kindly provided by the Brazilian Association for the Medicinal Use of *Cannabis* (ABRACAM; 257 Francisco Teixeira de Alcântara Street, Fortaleza, CE, Brazil). Oil extraction was performed using the Rick Simpson method. This technique involves the production of a concentrated *Cannabis* oil, also known as “Rick Simpson’s Oil” (RSO). The goal is to produce a highly concentrated oil containing a large amount of active *Cannabis* compounds, such as the cannabinoids THC and CBD [55].

The Nuclear Magnetic Resonance (NMR) profile for the crude oils, Cs-CBD and Cs-THC, both containing Hydrogen-1 (1H NMR) and Carbon-13 (13C NMR), were characterized using an Avance DRX 500 MHz Spectrometer (Bruker, Billerica, MA, USA) at CENAUREMN (Northeastern Center for the Application and Use of NMR, Department of Organic and Inorganic Chemistry-UFC, Fortaleza, Brazil). Cs-CBD was adsorbed on silica gel and subjected to preparative column chromatography to generate a fraction mainly composed of CBD, which was characterized by 1H NMR. The process was repeated once to provide a solid fraction called purified CBD (CBD-pur.), also characterized by 1H NMR and 13C NMR. Cs-CBD and CBD-pur. were compared by thin-layer chromatography. Likewise, Cs-THC was adsorbed onto silica gel and subjected to preparative column chromatography to generate a fraction composed mainly of THC. This fraction, referred to as purified THC (THC-pur.), was characterized by 1H NMR and 13C NMR, revealing a high degree of purity. The Cs-THC and THC-pur. were compared by thin-layer chromatography. Both Cs-CBD and CBD-pur., as well as Cs-THC and THC-pur. in nanoemulsion form, were pharmacologically tested in vivo.

### 4.2. Preparation of Nanoemulsions

The oil extracted from the nuts of licuri or ouricuri (*Syagrus coronata*), a palm tree from the Arecaceae family, was obtained from Licuri Brazil (Caldeirão Grande, BA, Brazil). The non-ionic surfactant used was the triblock copolymer poly(ethylene oxide)-poly(propylene oxide)-poly(ethylene oxide) (Pluronic^®^ F127) from Sigma-Aldrich, St. Louis, MI, USA. For the preparation and characterization of the nanoemulsions, as well as the characterization methods, all reagents were of analytical grade.

The fatty acid composition of licuri oil was assessed using gas chromatography coupled with mass spectrometry (GC-MS) on a SHIMADZU QP-2010 ULTRA instrument, Shimadzu, Kyoto, Japan, utilizing a (5%-phenyl)-methylpolysiloxane (DB-5) capillary column (30 m × 0.25 mm), with helium as the carrier gas (flow rate of 0.6 mL/min) in splitless mode (injection volume of 1 μL of 1 mg/mL solutions of each product diluted in ethyl acetate). The oven temperature was initially set at +120 °C and increased at 10 °C/min to +300 °C, then maintained for 10 min. The injector and detector temperatures were +250 and +300 °C, respectively. The quadrupole analyzer was set to electron ionization (EI) mode and scanned from 50 to 450 *m*/*z*.

The aqueous phase (AP) and the organic phase (OP) were prepared and homogenized separately. The amphiphilic surfactant Pluronic^®^ F127 (0.2 g) was added to the AP (90% of the formulation). Licuri oil (1 g) and THC (0.1 g) or CBD (0.1 g) constituted the OP (10% of the formulation). To produce each primary emulsion (10 g), the previously prepared AP was slowly added to OP and stirred at 1000 rpm for 30 min. To generate THC and CBD nanoemulsions, each primary emulsion was sonicated using a probe sonicator (Digital Sonifier W-450D, Branson, Marshall Scientific, Hampton, NH, USA) for 3 min (10 s on/10 s off), at 70% amplitude, in an ice bath.

The nanodroplet size, PDI, and ZP of THC and CBD nanoemulsions were determined using a dynamic light scattering (DLS) instrument at 25 °C with a Zetasizer (Malvern Instruments, Malvern, UK). The nanoemulsions were diluted at 1:1000 with deionized water. Averages and standard deviations were calculated in triplicate.

### 4.3. Animals and Treatments

Male Swiss mice (n = 15; body weight: 25–33 g) were kept at 25 ± 2 °C, under a 12/12-h light/dark cycle. Food and water were provided ad libitum. This study was evaluated and approved by the Ethics Committee on Animal Research of the Faculty of Medicine at Federal University of Ceará, protocol number 7839060721.

PTZ (99% purity) was purchased from Sigma-Aldrich (São Paulo, SP, Brazil). Depakene (solution containing 50 mg/mL sodium valproate) was purchased from Abbott Brazil Laboratories Ltd.a (São Paulo, SP, Brazil). All other drugs and reagents were of analytical grade.

Mice received either the vehicle (nanoemulsions without CBD or THC) or nanoemulsions containing CBD or THC, respectively, at doses 1, 3, 6, and 10 mg/kg, or the combination of CBD and THC (1:1 volume/volume) at 1.5, 3, and 6 mg/kg. Sixty minutes later, PTZ (80 mg/kg) was i.p. administered and animals were set in pairs at the center of plastic boxes, in a quiet environment. All experimental groups were visually monitored for one hour after the PTZ injection. The following parameters were measured: latency to first generalized seizure; latency to death; and survival rate. After recovery, surviving animals were kept in cages with chow and water to evaluate the survival rate after 24 h [56,57,58,59]. Brains were dissected for the neurochemical assays immediately after death.

The valproic acid control group was excluded from these tests because its action primarily targets neurotransmission, with little direct impact on oxidative and nitrosative stress markers. In this way, we optimized the comparison between cannabinoids and minimized the unnecessary use of animals. This decision follows the principles of the 3Rs (Reduce, Reuse, and Refine), seeking to minimize the number of animals used, avoid redundancy, and maximize scientific efficiency.

Further mice (n = 15 animals per group) received PTZ 30 mg/kg i.p. every other day for 21 days or until the development of full kindling, corresponding to the most severe seizure stage. Sixty minutes before PTZ administration, either vehicle, CBD 10 mg/kg or the CBD/THC (1:1) combination at 1.5, 3, and 6 mg/kg, or valproic acid 50 mg/kg were p.o. administered. On the 21st day of the protocol, after the PTZ injection, animals were scored over 30 min to grade behavioral seizures according to the Racine scale, consisting of the following: stage 1—normal behavior; stage 2—hyperactivity; stage 3—repeated vertical movements that may represent stereotypical behavior; stage 4—clonus of the forepaws and “rearing”, and stage 5—clonus of all paws, loss of righting reflex and fall, tonic ventral flexion of the head and the whole body, and tonic hindpaw extension [58]. Full kindling was considered when the animal presented a motor behavior corresponding to stage 5 [60]. Additionally, a group was treated solely with CBD/THC (1:1) at 6 mg/kg, without PTZ, to assess the effects of the cannabinoid combination independently. On the following day, behavioral tests were carried out before killing for immunohistochemistry.

### 4.4. Behavioral Analysis

The novel ORT is used to evaluate recognition memory. This task is based on the innate tendency of rodents to explore unfamiliar objects within their environment. This test assesses the mouse’s ability to discriminate between familiar and novel objects. Firstly, mice were individually habituated to an open field Plexiglas^®^ box (30 × 30 × 40 cm) for 5 min. After 15 min, mice were allowed to explore a set of two identical objects for 5 min (acquisition phase). These objects were suitably heavy and long to ensure that mice could neither displace them nor climb over them. After a 5 min interval, mice were presented to a similar set of objects in the same environment, with the replacement of one familiar object by a novel/unknown object (testing phase). The animals were allowed to freely explore the objects again for a 5 min long period. The discrimination index was calculated as follows: (time exploring new object-time exploring familiar object)/(time exploring new object + time exploring familiar object) [59,61].

Working memory and learning were assessed by the rate of spontaneous alternations in the Y-maze (40 × 5 × 16 cm), with three arms positioned at equal angles, as previously described [60]. Before running the test, the arms were numbered, and the animal was placed in one arm and allowed to spontaneously alternate entries into the other arms for 8 min. The sequence of the arms into which the animal entered was then noted down and the information analyzed to determine the number of arm entries without repetition [62].

### 4.5. Evaluation of Oxidation in the Brain

Animals that underwent the acute PTZ protocol had their brains used for neurochemical assays of NO^3−^/NO^2−^ and TBARS. For this purpose, prefrontal cortices, hippocampi, and striata were dissected on aluminum foil-covered ice. Brain areas were kept under −80 °C until use.

Griess reagent was added to a 96-well plate containing the supernatant of the homogenates of prefrontal cortices, hippocampi, and striata from 6–8 animals. The absorbance was measured using a microplate reader at 560 nm. Samples were harvested from animals treated with only vehicle (no PTZ, basal level group). Previously, a standard curve for nitrite was generated using concentrations of 100, 50, 25, 12.5, 6.25, 3.12, and 1.56 nmol/mL. Results were expressed as nmol/g tissue [63,64].

Brain areas (prefrontal cortex, hippocampus, and striatum) from 6–8 animals were used to prepare 10% homogenates in 1.15% KCl. Then, 250 μL were added to 1 mL 10% trichloroacetic acid, followed by the addition of 1 mL 0.6% thiobarbituric acid. After agitation, the mixture was kept in a water bath (95–100 °C, 15 min), cooled on ice, and centrifuged (1500× *g*/5 min). The TBARS content was determined in a plate reader at 540 nm, with results expressed in nmol of MDA per gram of tissue. A standard curve with MDA was performed previously [64].

### 4.6. Immunohistochemistry

Animals that underwent the PTZ-induced kindling protocol were used for immunohistochemistry after behavioral testing. The euthanasia protocol consisted of i.p. injections of ketamine (90 mg/kg) and xylazine (15 mg/kg). After the loss of withdrawal reflex to paw pinch, animals were transcardially perfused with 0.9% saline (about 60 mL) for vascular rinsing, followed by 60 mL of 4% paraformaldehyde solution in phosphate-buffered saline (pH 7.4). Brains were then post-fixed in the same 4% PFA solution at 4 °C for 24 h. After this, brains were immersed in 70% alcohol for subsequent paraffin embedding; 3 μm thick coronal slices were then obtained using a microtome (Leica, Wetzlar, Germany). Coronal sections were obtained from −2.46 mm to −2.92 mm (caudal to bregma), containing the hippocampus, and postfixed in 70% ethanol. After cooling, the sections were washed four times with PBS, and endogenous peroxidase was blocked with 3% H_2_O_2_ in PBS (15 min). Sections were incubated overnight (4 °C) with primary antibodies (anti-GAT-1 1:200, Abcam ab426; anti-GFAP 1:200, Thermo Fisher 53-9892-82; anti-KCNJ10 1:200, EAB-19262Elabscience, Houston, TX, USA) in PBS, according to the manufacturer’s instructions. On the next day, sections were washed in PBS four times, incubated (30 min) with secondary biotinylated rabbit antibody (anti-IgG) in PBS (1:200), washed four times in PBS, and incubated (30 min) with the conjugated streptavidin–peroxidase complex (Burlingame, CA, USA). After washing, sections were developed with 3,3-diaminobenzidine, mounted on gelatinized glass slides, dehydrated, and coverslipped for analysis [65]. The images were analyzed semi-quantitatively (ImageJ software, 1.8.0 version, NIH, Bethesda, MD, USA) using the following plugin: Color deconvolution HDAB-Threshold-Measure particle.

### 4.7. Statistical Analysis

Statistical analyses were performed using GraphPad Prism 8.0. The normality of the data was assessed using the Shapiro–Wilk test. Data with a normal distribution were expressed as mean ± standard error of the mean (SEM) and compared using one-way ANOVA followed by Tukey’s multiple comparison test. For data that did not meet normality assumptions, the Kruskal–Wallis test was applied, followed by Dunn’s multiple comparison test for post hoc analysis. Survival analysis during the PTZ-induced kindling protocol was conducted using the Log-rank test. A 95% confidence interval was adopted for all analyses, and *p*-values < 0.05 were considered statistically significant.

## 5. Conclusions

This study underscores the potential of CBD and THC nanoemulsions in seizure models, highlighting their capacity to reduce convulsions and brain damage. These formulations significantly decreased markers of oxidative and nitrosative stress, enhancing our grasp of their antiseizure mechanisms. Molecular and cellular changes, such as variations in the expression of GFAP (indicative of astrocyte response), GAT1 (a GABA transporter affecting neurotransmitter uptake), and Kir4.1 (a potassium channel involved in glial buffering), pave new paths for therapeutic interventions in epilepsy, beyond merely neurochemical alterations involving neurotransmitters, by encompassing broader aspects like receptor activity, structural changes, and glial proliferation.

Despite its promise, this study faces limitations due to the use of animal models, which may not fully replicate human epilepsy complexity. Further research is needed to validate these findings in clinical trials and to explore the efficacy and safety of CBD and THC nanoemulsions in a broader range of neurological disorders. Future prospects include optimizing nanoemulsion formulations for enhanced bioavailability and therapeutic efficacy, as well as deepening the study of underlying molecular mechanisms to develop more effective and safer epilepsy treatment strategies.

## Figures and Tables

**Table 1:** Fatty acid composition of licuri oil, compared to that reported in a previously published paper (* reference [25]).

Fatty Acid	Symbol	Peak Area (%)	Peak Area (%) *
capric	C 10:0	7.87	6.40
lauric	C 12:0	42.13	43.64
myristic	C 14:0	16.14	14.32
palmitic	C 16:0	9.18	6.89
oleic	C 18:1	19.52	11.78
stearic	C 18:0	5.15	3.83
caprylic	C 8:0	-	10.05
linoleic	C 18:2	-	3.1

**Table 2:** Characterization of Δ9-tetrahydrocannabinol (THC) and cannabidiol (CBD) nanoemulsions by polydispersity index (PDI) and zeta potential (ZP).

Formulation	Droplet Size (nm)	PDI	ZP (mV)
THC nanoemulsion	237.5 ± 1.46	0.09 ± 0.03	−33.9 ± 2.14
CBD nanoemulsion	247.2 ± 1.15	0.08 ± 0.02	−32.4 ± 1.37

## Data Availability

Data is contained within the article.
